# A Novel Dopingless Fin-Shaped SiGe Channel TFET with Improved Performance

**DOI:** 10.1186/s11671-020-03429-3

**Published:** 2020-10-17

**Authors:** Shupeng Chen, Shulong Wang, Hongxia Liu, Tao Han, Haiwu Xie, Chen Chong

**Affiliations:** School of Microelectronics, Key Laboratory of Wide Band-Gap Semiconductor Materials and Devices of Education, Xidian University, Xi’an, 710071 China

**Keywords:** Tunnel FET, Dopingless, Fin-shaped channel, Line tunneling junction, Stack gate oxide

## Abstract

In this paper, a dopingless fin-shaped SiGe channel TFET (DF-TFET) is proposed and studied. To form a high-efficiency dopingless line tunneling junction, a fin-shaped SiGe channel and a gate/source overlap are induced. Through these methods, the DF-TFET with high on-state current, switching ratio of 12 orders of magnitude and no obvious ambipolar effect can be obtained. High *κ* material stack gate dielectric is induced to improve the off-state leakage, interface characteristics and the reliability of DF-TFET. Moreover, by using the dopingless channel and fin structure, the difficulties of doping process and asymmetric gate overlap formation can be resolved. As a result, the structure of DF-TFET can possess good manufacture applicability and remarkably reduce footprint. The physical mechanism of device and the effect of parameters on performance are studied in this work. Finally, on-state current (*I*_ON_) of 58.8 μA/μm, minimum subthreshold swing of 2.8 mV/dec (SS_min_), average subthreshold swing (SS_avg_) of 18.2 mV/dec can be obtained. With improved capacitance characteristics, cutoff frequency of 5.04 GHz and gain bandwidth product of 1.29 GHz can be obtained. With improved performance and robustness, DF-TFET can be a very attractive candidate for ultra-low-power applications.

## Introduction

With the scaling down of MOSFETs, the switching speed, high-frequency performance, density, cost and functionality of integrated circuits (ICs) are meet a great improvement[1]. But with the continuous progress of voltage scaling down, the unacceptable high-power consumption becomes a serious problem for modern ICs [1, 2]. Benefit from the band-to-band tunneling mechanism, tunnel FET (TFET) with steep SS and low-power consumption bring a new solution to this problem and attracted lots of attention [3–9]. But the applications of conventional silicon-based TFETs are limited by the considerably low on-state current (*I*_ON_), low switching ratio, severe ambipolar effect and large average subthreshold swing (SS) [1, 7]. To improve the performance of TFETs, applications of new structures and new materials on TFETs have been proposed in recent years. For example, TFETs with tunneling rate enhanced layer are proposed in recent years [5, 10, 11]. With this layer, the effective length of tunneling path is reduced and results to an obvious tunneling rate enhancement. Moreover, TFETs with improved gate structure are studied by many research groups [12–20]. The concept of line tunneling is introduced in L-TFET [17–19]. As a result, SS_avg_ of 42.8 mV/decade and *I*_ON_ of 10^−6^A/μm can be achieved by L-TFET. To further improve the performance of TFETs, an improved TG-TFET with T-shaped overlap and dual source is reported [20, 21]. As a result, the *I*_ON_ of TG-TFET reaches 81 μA/μm. To further improve the device performance, high requirement on doping profile of the tunneling junction is required. Foundry engineers need to create a ultra-steep abrupt junction which has only several nanometers thick, and this is very difficult to achieve. In order to avoid this difficulty, the dopingless TFET (DL-TFET) on thin intrinsic semiconductor film using charge plasma concept is reported by research groups [22, 23]. In DL-TFET, the manufacture difficulty can be significantly reduced by removing the ultra-steep abrupt junction. The performance degradation induced by random dopant fluctuations can be avoided. Moreover, the fabrication of the DL-TFET does not demand high thermal budgets for creating the source and drain, which opens up the possibility of realizing TFETs on other substrates such as single crystal silicon on glass. As a result, the SS of DL-TFET has been greatly improved. However, due to the low efficiency of point tunneling junction, the current of DL-TFET is not high enough, which is difficult to meet the ever-increasing requirements of modern circuit applications.

In this paper, a novel dopingless fin-shaped SiGe channel TFET (DF-TFET) is proposed and studied. To improve performance and robustness of the device, line tunneling junction and SiGe material are applied to DF-TFET. Meanwhile, gate metal work function is optimized to further improve the tunneling rate. Moreover, the dopingless channel reduced the manufacture process difficulty while the fin structure makes the asymmetric gate/backgate manufacture applicable. As a result, on-state current (*I*_ON_) of 58.8μA/μm, off-state leakage current (*I*_OFF_) near 10^−11^ μA/μm, average subthreshold swing (SS_avg_) of 18.2 mV/dec and minimum subthreshold swing (SS_min_) of 2.8 mV/dec can be reached by DF-TFET. With relatively small gate capacitance (*C*_gg_) and gate to drain capacitance (*C*_gd_), good analog/RF performance can be obtained. Finally, the cutoff frequency (*f*_T_) reached 5.04 GHz and gain bandwidth product (GBW) reached 1.29 GHz.

The structures of this paper are as follows: “Device Structure and Simulation Method“ section shows the TCAD simulation methods of this work. The structure and the parameter of DF-TFET are introduced. The differences and advantages of DF-TFET compared with DL-TFET and TG-TFET are illustrated. The mechanism, characteristic and analog/RF performance of DF-TFET are discussed in “Results and Discussion” section. A performance comparison between DF-TFET, DL-TFET and TG-TFET is carried out in this section. Moreover, the influence of the device parameters on performance and DF-TFET’s typical RF parameters is studied in this section. In order to further understand the potential of DF-TFETs in ultra-low-power applications, comparisons of electrical characteristics among different TFETs and DF-TFETs under low voltage bias were carried out.

## Device Structure and Simulation Method

**Fig. 1:**
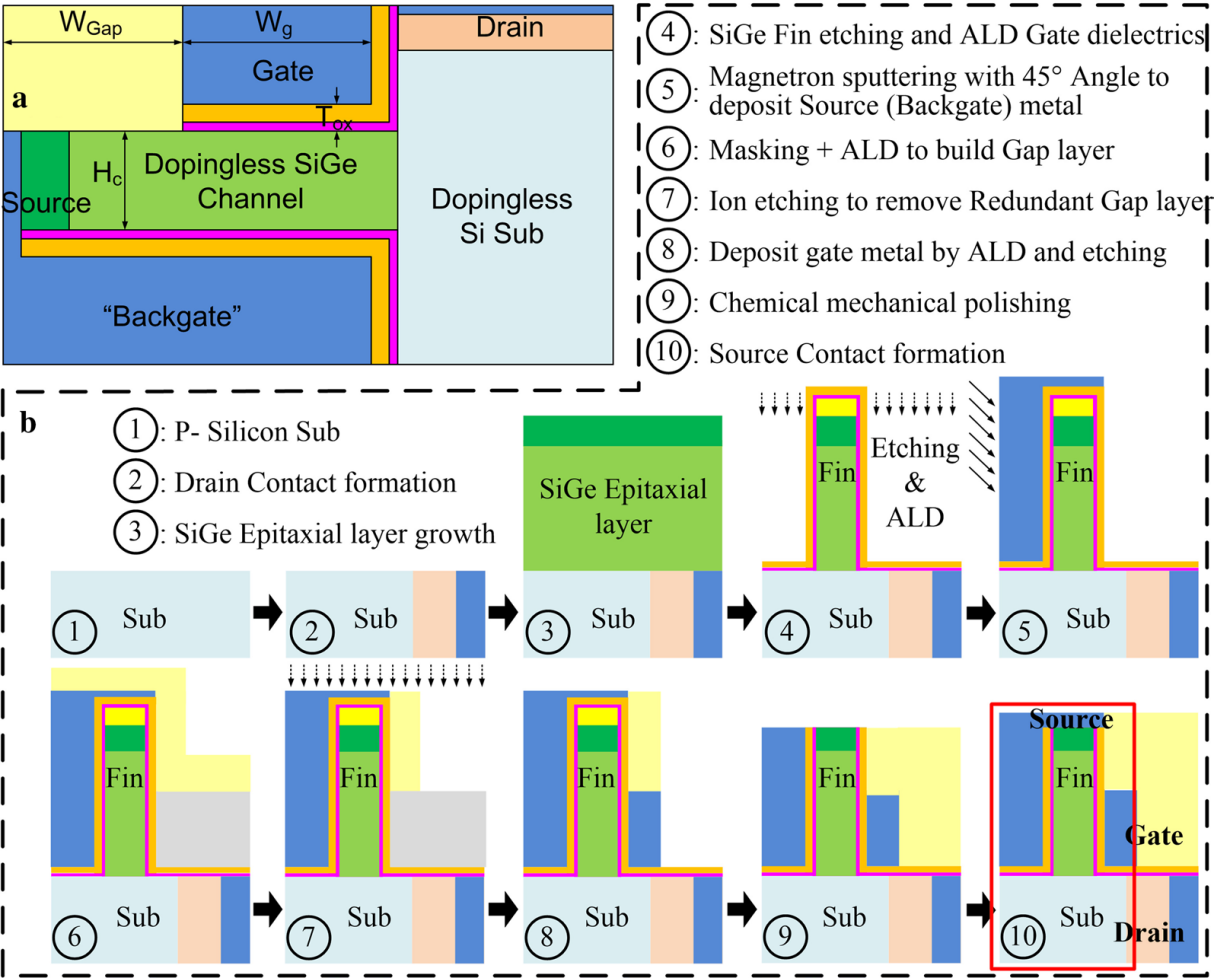
**a** Structure and **b** fabrication flow of DF-TFET

The proposed structure of DF-TFET is illustrated in Fig. 1a. To improve the device performance, line tunneling junction is applied to the dopingless fin-shaped SiGe channel by charge plasma concept [24, 25]. It is known that gate dielectric thickness can significantly affect the tunneling current. This is because in the result of the WKB approximation [26], as shown in Eq. (1), the tunneling probability depends on effective screening length (*λ*), effective carrier mass (m^*^), energy band gap (*E*_g_) and effective screening energy window (Δ*Φ*).

1 $$T_{{{\text{WKB}}}} \approx \exp \left( { - \frac{{4\lambda \sqrt {2m^{ * } } \sqrt {E_{{\text{g}}}^{3} } }}{{3q\hbar (E_{{\text{g}}} + \Delta \Phi )}}} \right)$$

Reducing the thickness of the gate dielectric or using a high *κ* dielectric will reduce *λ* and increase Δ*Φ*, which will cause a tunneling probability increase exponentially. Thus, using high *κ* dielectrics and reducing the dielectric thickness can significantly increase the on-state current of TFET. But the small dielectric thickness and interface quality issues of high *κ* dielectrics will cause serious reliability problems. Thus, a stack gate dielectric of 0.5 nm of Al_2_O_3_ and 2.0 nm of HfO_2_ is set to guarantee a good interface quality [27–29], which can significantly reduce the leakage current and improve the reliability of gate dielectric. The source electrode is located on the top of the fin structure. At the same time, it is also next to one side of the fin and works as a “backgate” to apply a zero bias. By using gate and source electrode with different metal work functions, a line tunneling junction can be formed in the dopingless fin-shaped SiGe channel by charge plasma concept. The band-to-band tunneling (BTBT) direction is perpendicular to the channel/gate surface. This can help increase *I*_ON_ by improving the effective tunneling junction area.

High switching ratio (*I*_ON_/*I*_OFF_) can be obtained due to the large on-state current (*I*_ON_) and small off-state current (*I*_OFF_) provided by the line tunneling junction. Furthermore, the application of a fin structure in DF-TFET can remarkably reduce the footprint compared to the planer line tunneling TFET [30, 31]. Figure 1b shows an available fabrication flow to form the structure of DF-TFET. Table 1 shows the main process parameters of DF-TFET. Finally, without the difficulty to make a steep and uniform abrupt p–n junction, good device performance and robustness can be achieved.

**Table 1:** Main parameters of DF-TFET

Parameter	DF-TFET
Length of gate (*W*_g_)	30 nm
Length of gap (*W*_Gap_)	15 nm
Stack gate oxide thickness (*T*_ox_)	2.5 nm
Channel thickness (*H*_c_)	5 nm
Gate/drain work function ( *φ*_Gate_/*φ*_Drain_)	5.1 eV
Ohmic contact doping concentration	1 × 10^20^ cm^−3^

**Fig. 2:**
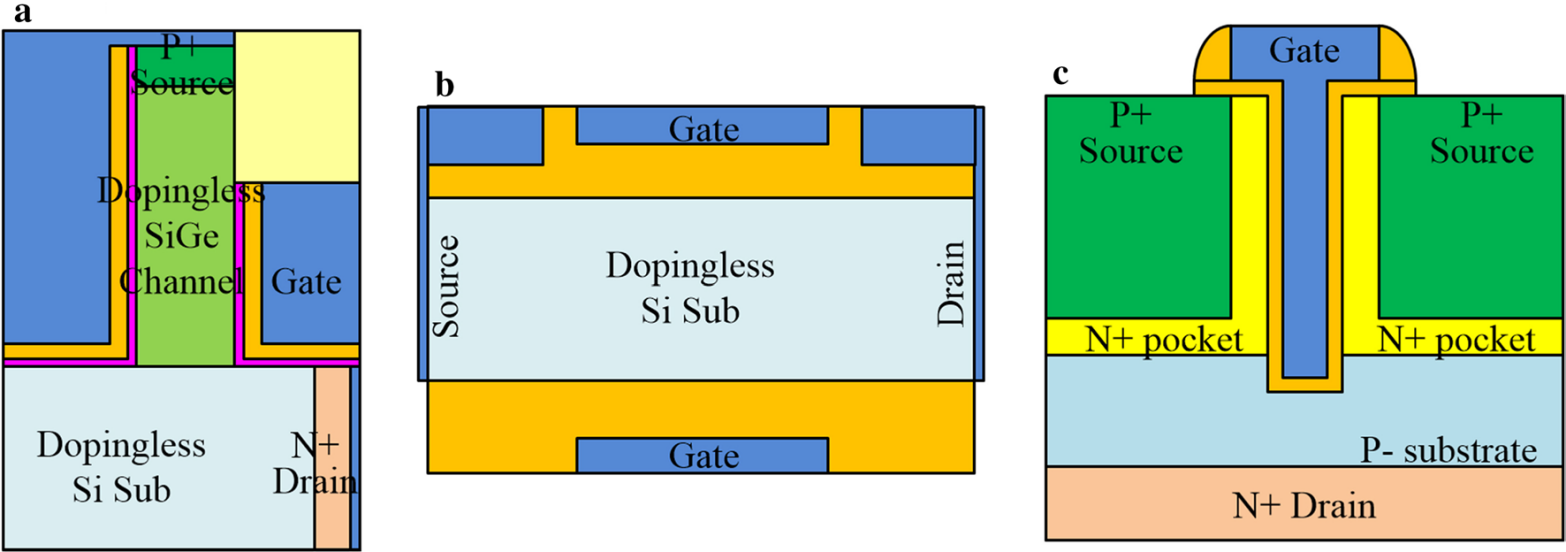
Structure of **a** DF-TFET, **b** DL-TFET, **c** TG-TFET

To better understand of the differences and advantages of DF-TFET, DL-TFET and TG-TFET, Fig. 2 shows the structure of these three devices. With line tunneling junction, L-TFET and TG-TFET expected to obtain high on-state current. But experimental results show that the actual performance of L-TFET is not as high as expected [18, 19]. One of the most important reasons is the difficulty on form a steep and uniform abrupt p–n junction with perfect interface characteristics. Based on the structure of L-TFET, TG-TFET makes a great improvement on *I*_ON_. But TG-TFET is still facing the difficulty on forming a perfect abrupt p–n junction. Thus, to obtain the desirable good performance, a steep and uniform abrupt p–n junction which has only several nanometers thick should be obtained, but it is very difficult to realize in the manufacture process. By using a dopingless channel, DL-TFET can avoid this problem and bring better interface quality near tunneling junction. However, compared to line tunneling TFETs [16–21] with abrupt p–n junctions, simulation result shows that the *I*_ON_ of DL-TFET is relatively low [22, 23]. For further improvement, the DF-TFET is proposed and studied in this work.

Simulation of DF-TFET is carried out in Silvaco Atlas TCAD tools. Non-local BTBT model is introduced in this simulation to bring the energy band spatial variation into account, which can help to improve the accuracy of the BTBT tunneling process. Lombardi mobility model is considered to make the channel mobility accurate. Band-gap narrowing model is taken into account to fit the heavy doped ohmic contact regions, and Shockley–Read–Hall recombination model is taken into consideration in this paper, too.

## Results and Discussion

### Mechanism and Comparison of DF-TFET, DL-TFET and TG-TFET

**Fig. 3:**
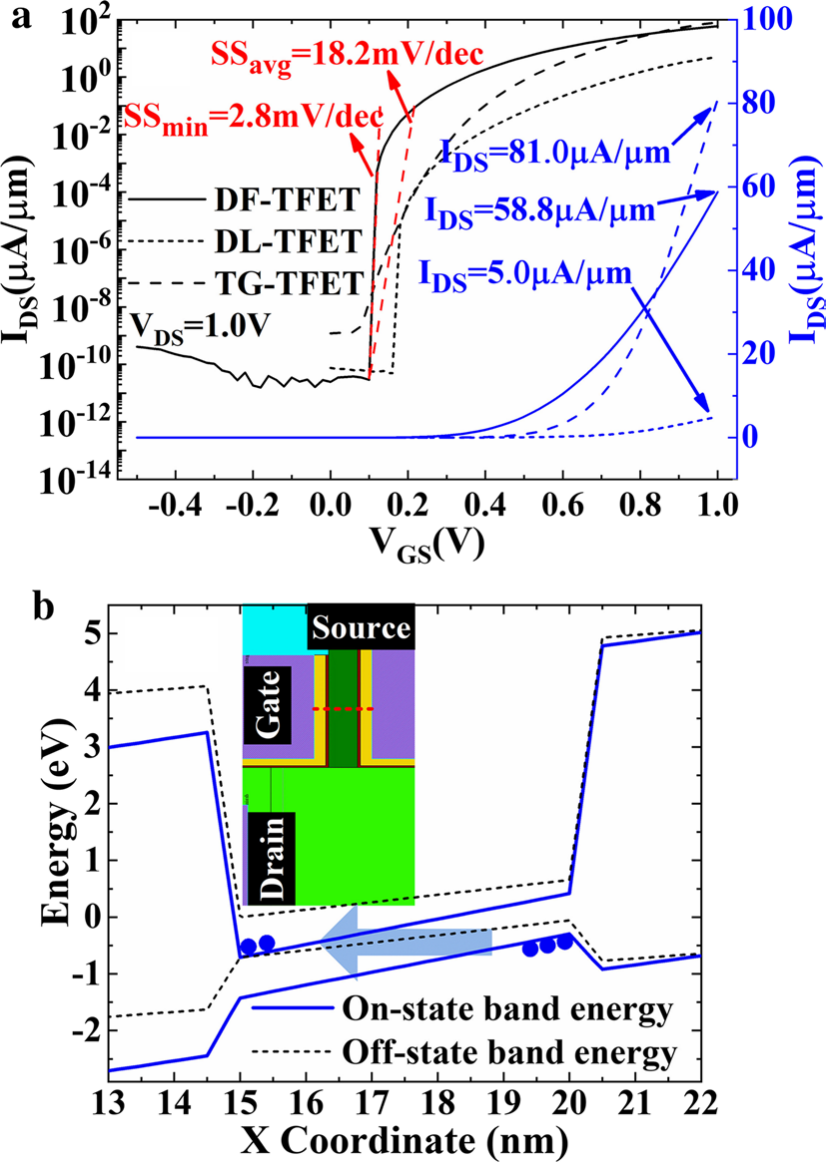
**a** Transfer characteristics of DF-TFET, DL-TFET and TG-TFET. **b** Energy band condition of DF-TFET in different work conditions (the inset shows the cut line position)

Figure 3a shows the transfer characteristics comparison of DF-TFET, DL-TFET and TG-TFET. Benefit from the line tunneling junction in the fin-shaped SiGe channel, DF-TFET reaches an on-state current (*I*_ON_) of 58.8 μA/μm and achieves a large switching ratio of over 12 orders of magnitude where no obvious ambipolar effect occurs. Furthermore, minimum subthreshold swing (SS_min_) of 2.8 mV/dec and average subthreshold swing (SS_avg_) of 18.2 mV/dec are obtained. As a result, DF-TFET has obvious improvement in *I*_ON_ compared to DL-TFET and subthreshold swing compared to TG-TFET. *I*_ON_ of DF-TFET is more than one order of magnitude larger than DL-TFET at *V*_DS_ = *V*_GS_ = 1 V. Figure 3b shows the energy band condition of DF-TFET and illustrates the formation of tunneling window in fin-shaped channel. The red dotted line in the inset of Fig. 3b shows the position where the energy band curve is obtained.

**Fig. 4:**
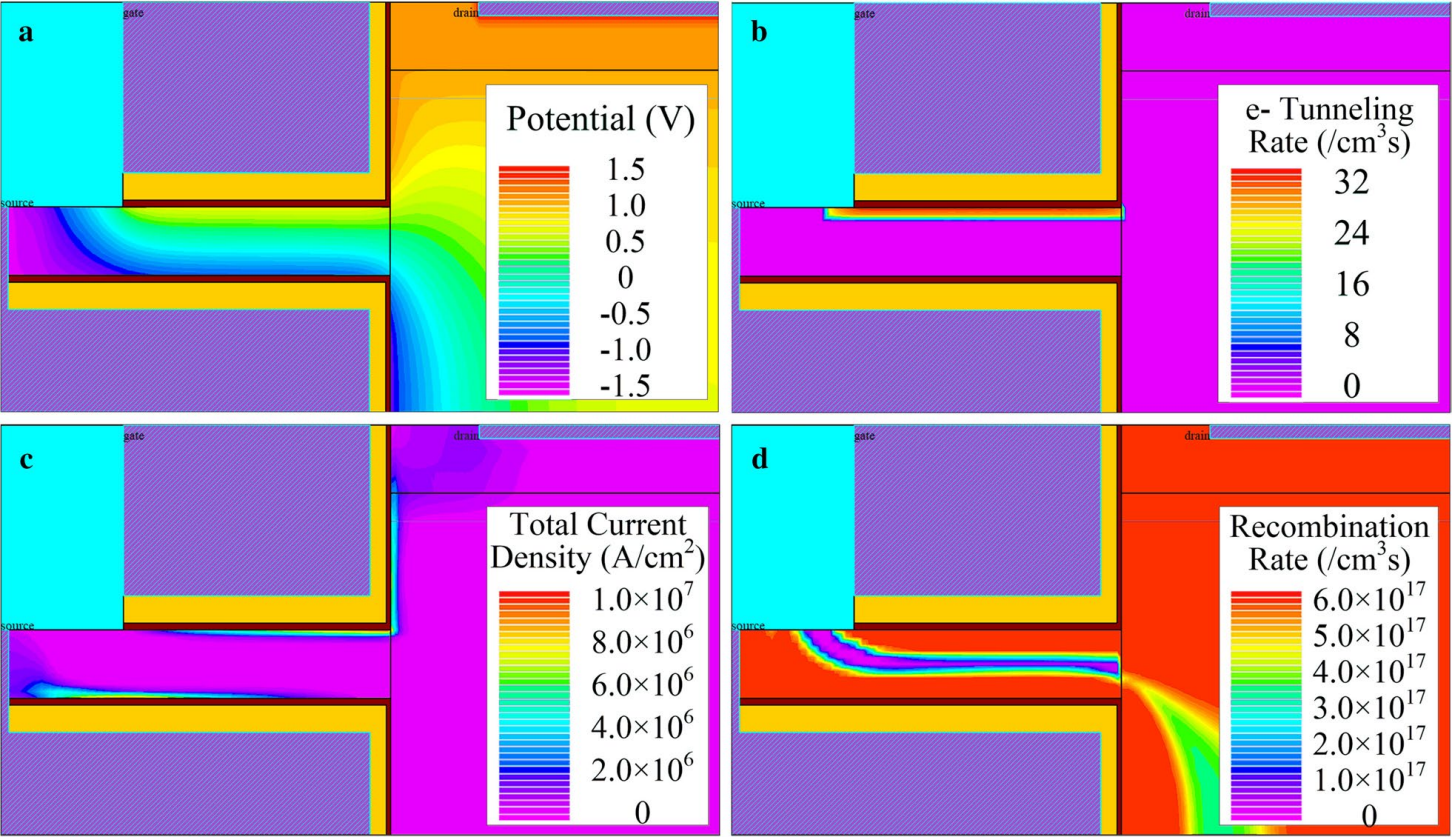
Distribution of **a** potential, **b** e tunneling rate, **c** current density and **d** recombination rate of DF-TFET

The distribution of important physical quantity in DF-TFET’s tunneling process is shown in Fig. 4, which includes the distribution of (a) potential, (b) e tunneling rate, (c) total current density and (d) recombination rate in an on-state work condition. In Fig. 4a, a clear potential gradient perpendicular to the gate/channel interface can be observed. Thus, a huge potential difference is generated in fin-shaped channel and this will modulate the concentration of electrons and holes on both sides of fin channel. At the same time, a steep energy band bending can be formed in the fin-shaped channel. As a result, a line tunneling junction parallel to the gate/channel interface can be formed. Figure 4b shows the e-tunneling rate in the fin structure channel. The peak value of e-tunneling rate is uniformly distributed near the gate/channel interface and parallel to the surface. This proves that the line tunneling junction is parallel to the gate/channel interface. Figure 4c shows the current path in DF-TFET. The valence band electrons from the backgate/channel side are tunneling to the conduction band near the gate/channel side. Under the influence of gate voltage and drain voltage, electrons move along the fin channel to the drain electrode. In this way, a tunneling current path is formed in DF-TFET. Figure 4d shows the recombination rate distribution in DF-TFET; this can illustrate the location of the tunneling junction more obviously. The purple strip in the SiGe fin channel can represent the location of the tunneling junction.

### DC Characteristics with Different Parameters and Analog/RF Performance

**Fig. 5:**
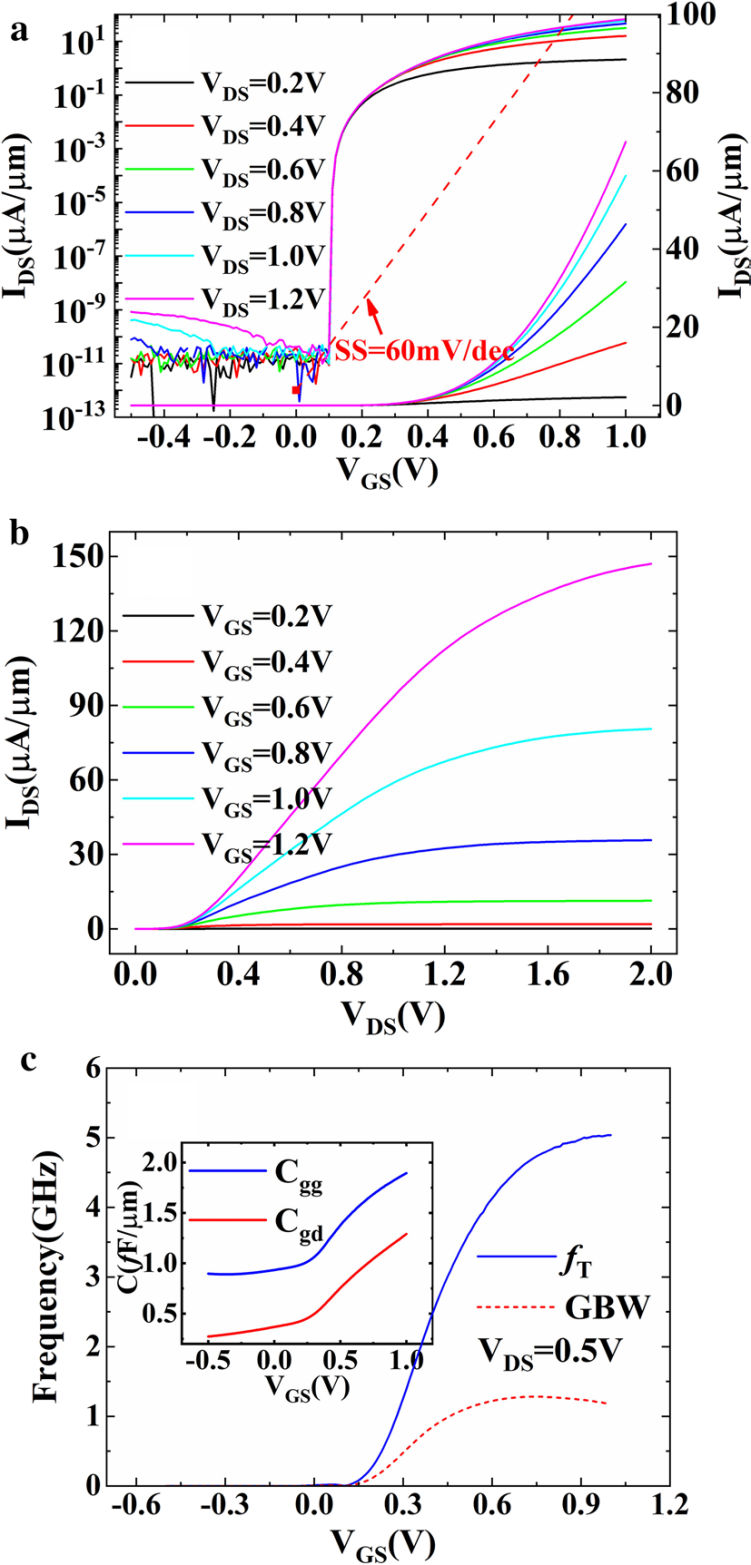
**a** Input, **b** output and **c** radio frequency characteristics of DF-TFET

Figure 5a, b shows the input and output characteristics of DF-TFET under different biases. The increasing of *V*_DS_ has little effect on subthreshold swing characteristics, but *I*_ON_ will have a linear growth while *V*_DS_ increases from 0.2 to 1.2 V (at *V*_GS_ = 1.0 V). Figure 5c shows the cutoff frequency (*f*_T_) and gain bandwidth product (GBW) calculated by Eqs. (2) and (3). Result shows that a cutoff frequency of 5.04 GHz and a gain bandwidth product of 1.29 GHz can be obtained.

2 $$f_{{\text{T}}} = \frac{{g_{{\text{m}}} }}{{2\pi C_{{{\text{gs}}}} \sqrt {1 + 2C_{{{\text{gd}}}} /C_{{{\text{gs}}}} } }} \approx \frac{{g_{{\text{m}}} }}{{2\pi \left( {C_{{{\text{gs}}}} + C_{{{\text{gd}}}} } \right)}} = \frac{{g_{{\text{m}}} }}{{2\pi C_{{{\text{gg}}}} }}$$

3 $${\text{GBW}} = g_{{\text{m}}} /2\pi 10C_{{{\text{gd}}}}$$

**Fig. 6:**
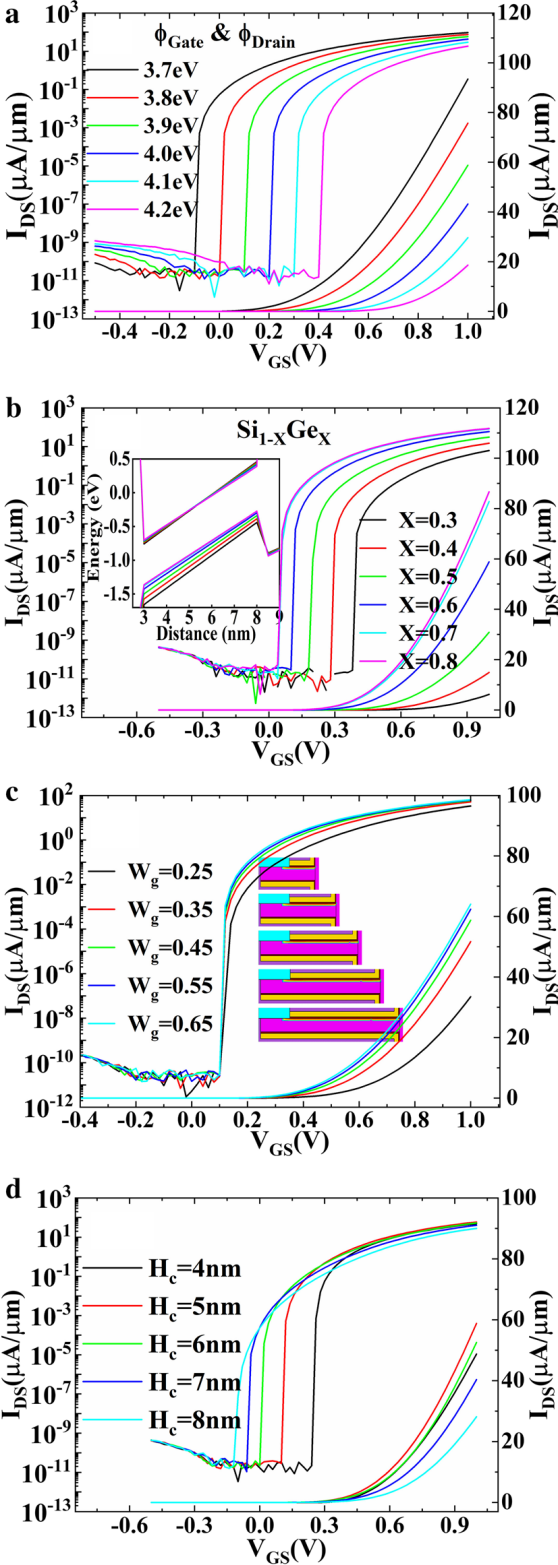
Transfer characteristics with different **a** gate work function (*φ*_Gate_) and drain work function (*φ*_Drain_), **b** SiGe composition ratio, **c** gate length (*W*_g_) and **d** channel thickness (*H*_c_)

Figure 6a shows the effect of gate work function (*φ*_Gate_) and drain work function (*φ*_Drain_) on transfer characteristics of DF-TFET. With the increasing work function, the transfer characteristic curve shifts toward the positive direction. As the work function varies from 3.7 to 4.2 eV, the *V*_th_ increases linearly from 0 to 0.5 V while the *I*_ON_ decreases linearly from 93.4 to 18.6 μA/μm. This makes it possible to adjust *V*_th_ to apply to different application requirements. Figure 6b shows the effect of composition ratio *X* of Si_1−*X*_Ge_*X*_. The increase in germanium composition leads to the decreasing of energy band gap and the increasing of tunneling window, as shown in the inset of Fig. 6b. Finally, results in the *I*_ON_ increase and transfer characteristic curve translates toward the negative direction. However, when *X* > 0.7, both the transfer characteristic curve and the *I*_ON_ have little change with the increasing *X*. This is because the channel energy band structure becomes insensitive to *X* when *X* > 0.7, as shown in Fig. 6b inset. Figure 6c, d shows the effect of gate length (*W*_g_) and channel thickness (*H*_c_) on transfer characteristics. The inset of Fig. 6c shows the dimensions of device channel under different *W*_g_. It is not difficult to observe from Fig. 6d that DF-TFET will suffer *I*_ON_ decrease when *H*_c_ becomes both too small and too large. Thus, a proper *H*_c_ will benefit the device performance.

In order to understand the potential of DF-TFET in ultra-low-power applications, Table 2 shows a performance comparison of different TFETs with DF-TFET. Compared to TFETs with a traditional heavily doped p–n tunneling junction [6, 20, 32–35], DF-TFET has obvious advantages on SS and switching ratio. This is due to the characteristics of DF-TFET by using electrostatically doping. Compared to other dopingless TFETs [22, 23, 36, 37], DF-TFET has obvious advantages on I_ON_. This is because of the improved tunneling rate by using line tunneling junction and SiGe material. By combining the advantages of p–n tunneling junction and dopingless tunneling junction, DF-TFET can provide high operating current and low static power consumption in ultra-low-power applications.

**Table 2:** Performance comparison of different TFETs with DF-TFET (at *V*_GS_ = *V*_DS_ = 0.5 V)

Device	*I*_ON_ (μA/μm)	Switching ratio	SS_avg_ (mV/dec)
V-TFET [32]	8	~ 10^7^	51
DS-TFET [33]	0.01	5.3 × 10^7^	64
SUTFET [6]	13.5	4.4 × 10^6^	25
TMG TFET [34]	10	3.3 × 10^8^	44
GS-TFET [35]	0.1	5.9 × 10^11^	40
TG-TFET [20]	0.6	6 × 10^8^	45
DL-TFET [22]	0.01	1.1 × 10^9^	51
ML-DL-TFET [23]	0.1	~ 10^13^	19
DMD-DLTFET [36]	5 × 10^–3^	~ 10^9^	43
H-DLTFET [37]	0.9	~ 10^14^	16 mV/de
This work	3.8	1.4 × 10^11^	18

## Conclusion

In this work, a novel DF-TFET is proposed and the electrical characteristics are analyzed by simulation method. The structure characteristic, physical mechanism, performance with different parameters and analog/RF performance of DF-TFET are discussed and studied. Benefit from the dopingless fin structure channel, stack gate dielectric, SiGe channel material and high-efficiency line tunneling junction, good performance in switching characteristics and analog/RF characteristics can be obtained. Moreover, by avoiding the formation of the abrupt p–n junction in manufacture process, uniform doping with high consistency and high robustness on process fluctuation can be achieved. Simulation result shows that, *I*_ON_ of 58.8 μA/μm, switching ratio of 12 orders of magnitude, no obvious ambipolar effect, SS_min_ of 2.8 mV/dec and *f*_T_ of 5.04 GHz can be achieved by DF-TFET. With the large operating current, high switching ratio, steep SS, good reliability, stable fabrication process and good manufacturability, it can be expected as one of the promising candidates for the future low-power IC and sensitive sensor applications.

## Data Availability

Not applicable (This manuscript is a purely theoretical study on analog/RF performance of TFET. The simulation data have been given in this manuscript, and it is not to be described here).

## Abbreviations

DF-TFET: Dopingless fin-shaped SiGe channel TFET
ICs: Integrated circuits
TGTFET: T-shaped gate dual-source TFET
DL-TFET: Dopingless TFET
*I*_ON_: On-state current
*I*_OFF_: Off-state current
SS_min_: Minimum subthreshold swing
SS_avg_: Average subthreshold swing
*C*_gg_: Gate capacitance
*C*_gd_: Gate to drain capacitance
*f*_T_: Cutoff frequency
GBW: Gain bandwidth product
*λ*: Effective screening length
*m*^*^: Effective carrier mass
*E*_g_: Energy band gap
Δ*Φ*: Effective screening energy window
*W*_g_: Length of gate
*W*_Gap_: Length of gap
*T*_ox_: Stack gate oxide thickness
*H*_c_: Channel thickness
*φ*_Gate_/*φ*_Drain_: Gate and drain work function
*V*_DS_: Drain to source voltage
*V*_GS_: Gate to source voltage
